# When Platelet Stimulation Becomes Marrow Stress: Rethinking Thrombopoietin Receptor Agonist Intensification in Pediatric Immune Thrombocytopenia

**DOI:** 10.3390/pediatric18030082

**Published:** 2026-06-17

**Authors:** Maurizio Aricò

**Affiliations:** Interdisciplinary Department of Medicine, University of Bari Aldo Moro, 70121 Bari, Italy; maurizio.arico@uniba.it

**Keywords:** immune thrombocytopenia, pediatric hematology, thrombopoietin receptor agonists, myelofibrosis, eltrombopag, romiplostim, avatrombopag, refractory ITP, treatment intensification, drug safety

## Abstract

Thrombopoietin receptor agonists (TPO-RAs) have become central second-line treatments for children with persistent or chronic immune thrombocytopenia (ITP). Their efficacy has encouraged broad use, but difficult-to-treat patients who respond suboptimally may be exposed to repeated agent switching, prolonged treatment, or doses exceeding approved limits. This commentary uses a focused narrative approach to address whether the risk of treatment-associated marrow fibrosis should be interpreted primarily as a consequence of treatment duration or as a risk marker linked to supraphysiological treatment intensity in non-responders. A recent Haematologica report by Ma and colleagues identified clinically significant bone marrow myelofibrosis in a highly selected cohort of children with chronic ITP undergoing marrow re-evaluation after suboptimal response or loss of efficacy during TPO-RA therapy. The most relevant message is not simply that fibrosis can occur, but that it was independently associated with treatment intensification, particularly overdose and frequent switching. The biological plausibility of this association is supported by the known capacity of sustained megakaryocytic stimulation to promote local pro-fibrotic signaling and reticulin deposition. This commentary places this safety concern in the context of pediatric ITP epidemiology, current regulatory indications, expert approaches to refractory disease, and practical surveillance considerations. TPO-RAs should not be viewed as routine treatment for newly diagnosed pediatric ITP; their principal role remains in selected children with persistent or chronic disease who require second-line therapy. Failure to respond at the maximum approved dose should prompt diagnostic and therapeutic reassessment rather than automatic treatment escalation. The emerging lesson is that response-adapted therapy must also be risk-adapted therapy.

## 1. Introduction

Thrombopoietin receptor agonists (TPO-RAs) have transformed the management of persistent and chronic immune thrombocytopenia (ITP) in children. By stimulating megakaryopoiesis, agents such as eltrombopag, romiplostim and avatrombopag offer an effective steroid-sparing strategy for patients in whom bleeding risk, impaired quality of life or treatment dependence make second-line therapy necessary [1,2,3,4,5,6]. Their introduction has therefore represented a major therapeutic advance in pediatric ITP.

However, the increasing confidence with which these agents are used has also created a new clinical problem: what should clinicians do when a child does not respond adequately? In daily practice, a suboptimal platelet response may prompt dose escalation, prolonged exposure or sequential switching between different TPO-RAs. Such decisions are often made under pressure, especially in children with clinically relevant bleeding, parental anxiety or limited therapeutic alternatives. Yet this is precisely the setting in which the bone marrow may be exposed to sustained supraphysiological stimulation.

## 2. Methodological Approach and Research Question

This article is a focused narrative commentary rather than a systematic review. The manuscript was developed around the following clinical question: in children with persistent or chronic ITP treated with TPO-RAs, should the risk of treatment-associated myelofibrosis be interpreted mainly as a function of cumulative exposure duration, or as a risk marker linked to supraphysiological treatment intensity in patients with suboptimal response? To address this question, the commentary integrates the findings of Ma et al. with selected peer-reviewed literature on pediatric ITP management, TPO-RA safety, marrow fibrosis biology, and refractory disease, together with official regulatory and demographic sources. The aim is not to establish new evidence-based surveillance guidelines, but to translate an emerging safety concern into a cautious clinical framework for risk interpretation and future study.

## 3. Population at Risk and Current Indications

A further distinction is clinically important before estimating the size of the population at risk. The children potentially exposed to prolonged TPO-RA therapy are not the broad group presenting with newly diagnosed, formerly acute, ITP. Current terminology classifies ITP according to duration as newly diagnosed during the first 3 months, persistent between 3 and 12 months, and chronic beyond 12 months from diagnosis [1]. In children with newly diagnosed ITP, management is usually based on observation when bleeding is absent or minor, and on short-course corticosteroids, intravenous immunoglobulin or anti-D immunoglobulin when treatment is required [2,3]. TPO-RAs are therefore not routine first-line agents for acute or newly diagnosed pediatric ITP. Their place is mainly in second-line management of persistent or chronic disease, particularly when bleeding symptoms, impaired quality of life or steroid dependence justify ongoing therapy [1,2,4,5,6].

This is consistent with European labelling: eltrombopag is indicated in children aged at least 1 year with primary ITP lasting 6 months or longer and refractory to other treatments, whereas romiplostim is indicated in children aged at least 1 year with chronic primary ITP refractory to other treatments [7,8]. Avatrombopag currently has an EMA indication for chronic ITP in adults, although the FDA label now includes pediatric patients aged at least 1 year with persistent or chronic ITP who have had an insufficient response to previous treatment [9,10]. Thus, while exceptional off-label use in severe early ITP cannot be categorically excluded, the safety concern raised here primarily concerns children with persistent or chronic, difficult-to-treat diseases.

## 4. Approach to Population Estimates

Although chronic ITP is rare in childhood, its absolute burden is not negligible. Pediatric ITP has been reported to occur at approximately 1.9–6.4 cases per 100,000 children per year, while chronic ITP has been estimated at about 0.5 per 100,000 children per year [11,12]. Using children aged 0–14 years as a conservative and readily available demographic denominator, the EU-27 population of 450.6 million, with 14.4% aged 0–14 years, corresponds to approximately 64.9 million children [13]. In Italy, the population aged 0–14 years is approximately 7 million [14]. On this basis, a chronic pediatric ITP frequency of 0.5 per 100,000 children per year would translate into roughly 325 new chronic pediatric ITP cases annually in the EU-27 and about 35 annually in Italy. If prevalence is approximated as incidence multiplied by mean disease duration, then average chronic disease durations of 3, 5, and 10 years would correspond to approximately 970, 1620, and 3240 prevalent children in the EU-27, and approximately 105, 175, and 350 in Italy, respectively (Table 1). These estimates are necessarily approximate, but they underline that even uncommon long-term treatment toxicities may become clinically relevant at the population level.

## 5. Treatment Intensification and Marrow Fibrosis

The recent Haematologica report by Ma and colleagues is therefore highly relevant [15]. In a retrospective cohort of 54 pediatric patients with chronic ITP who had received TPO-RA therapy for at least six months and underwent bone marrow biopsy because of suboptimal response or loss of efficacy, clinically significant myelofibrosis, defined as MF grade ≥ 2, was identified in 14 patients, corresponding to 25.9% of this highly selected population [15]. Importantly, this figure should not be interpreted as the expected prevalence among all children receiving TPO-RAs. Rather, it describes a difficult-to-treat referral cohort in whom marrow reassessment was clinically indicated. The clinical context of re-evaluation is also important: in the original study, treatment intensification, including dose escalation or switching, was typically prompted by initial suboptimal response, defined as failure to achieve a platelet count ≥30 × 10^9^/L and at least a two-fold increase from baseline within one month of a stable dose, or by subsequent loss of response, defined as a platelet decline to <30 × 10^9^/L after an initial response or recurrence of clinically significant bleeding [15].

The key observation is not simply that marrow fibrosis occurred, but that it was associated with treatment intensification. The term TPO-RA overdose in Ma et al. was operationally defined as any dose exceeding the maximum recommended pediatric weight-based dosage for each agent: eltrombopag > 1.5 mg/kg/day, avatrombopag > 1 mg/kg/day, hetrombopag > 0.15 mg/kg/day, and romiplostim > 10 µg/kg weekly [15]. This definition should therefore be interpreted as exposure beyond prespecified pediatric dose thresholds, rather than as accidental overdose. Among the 20 patients who met this definition, the median maximum administered dose was 168.5% of the recommended upper limit, and the median duration of overdose exposure was 7.8 months [15]. In multivariable analysis, TPO-RA overdose and the number of TPO-RA switches emerged as the only independent predictors of MF grade ≥ 2. TPO-RA overdose was associated with an adjusted odds ratio of 4.41 for clinically significant fibrosis, while each additional switch was associated with an adjusted odds ratio of 4.00. Time-to-event analysis reinforced this message: overdose was associated with accelerated development of MF ≥ 2, with an adjusted hazard ratio of 4.63 [15].

This is a clinically useful reframing. The traditional concern has often been cumulative duration of exposure: how long can a child safely remain on a TPO-RA? Ma et al. [15] suggest that the more relevant question may be: how intensely is the marrow being stimulated, and why? In children who respond to approved doses, long-term therapy may remain a reasonable and manageable strategy with appropriate monitoring. In contrast, repeated escalation beyond approved limits, especially when combined with switching between agents in search of a response, may identify a biologically and clinically different situation.

## 6. Clinical Interpretation

This distinction matters because TPO-RA failure is not merely a pharmacologic inconvenience. It may be a sign that the disease biology, diagnosis or therapeutic strategy requires reconsideration. This approach is consistent with expert recommendations for refractory ITP, which emphasize critical reassessment of the diagnosis and exclusion of non-autoimmune or secondary causes of thrombocytopenia before moving through further treatment tiers [16]. A child who remains thrombocytopenic despite maximum approved dosing should not automatically be subjected to prolonged off-label intensification. Instead, such a patient should prompt reassessment: confirmation of the ITP diagnosis, exclusion of inherited thrombocytopenias or marrow disorders, review of adherence and drug interactions, and consideration of alternative immunomodulatory approaches [1,2,16,17,18,19].

Another important point from the study is that marrow fibrosis may not be clinically obvious. At the time of biopsy, Ma et al. observed no significant differences in platelet count, white blood cell count, neutrophil count or hemoglobin between patients with MF 0–1 and those with MF ≥ 2; no teardrop cells were reported on peripheral blood smears [15]. This underlines the limitation of relying only on routine blood counts or smear morphology to detect clinically meaningful marrow reticulin or fibrosis in this setting.

The reversibility signal is also noteworthy, although necessarily preliminary. Among patients with MF ≥ 2 who underwent follow-up marrow evaluation, fibrosis regressed in those who discontinued TPO-RA therapy and transitioned to alternative immunomodulatory treatment, whereas persistence of MF ≥ 2 was observed in those who continued TPO-RA therapy [15]. This observation is consistent with the concept that TPO-RA-associated fibrosis may be at least partly reversible, but also that continued stimulation may maintain the fibrotic process [20,21,22].

## 7. Biological Plausibility and Possible Mechanisms

The association described by Ma et al. is also biologically plausible. TPO-RAs activate the thrombopoietin receptor pathway and expand megakaryopoiesis. In a physiologic or label-concordant therapeutic range, this effect is the basis of their clinical efficacy. However, sustained supraphysiological stimulation may amplify megakaryocyte proliferation and alter the marrow microenvironment. Megakaryocytes and platelets are important sources of pro-fibrotic mediators, including transforming growth factor-beta and other cytokines that can stimulate stromal cells and promote reticulin deposition [20,21,22,23,24]. This mechanism is conceptually consistent with the broader biology of myelofibrosis, in which abnormal megakaryocytic activity contributes to remodeling of the hematopoietic niche.

The adult ITP and TPO-RA safety literature has generally described marrow reticulin increase as an uncommon but recognized on-target phenomenon, often low grade and potentially reversible after dose reduction or drug discontinuation [20,21]. The morphology update by Harper et al. further illustrates that thrombopoietin mimetic-associated marrow fibrosis is an identifiable clinicopathologic phenomenon, while emphasizing that clinically significant fibrosis with collagen deposition is rare and that regression has been reported after withdrawal in selected cases [22]. Ma et al. extend this concept to a pediatric high-risk setting by suggesting that the relevant driver may not be exposure alone, but sustained marrow stimulation in children whose thrombocytopenia remains insufficiently responsive [15]. This does not prove a direct causal pathway in every patient, but it provides a coherent rationale for avoiding prolonged above-label stimulation and for reassessing diagnosis and strategy when response remains inadequate.

## 8. Limitations of the Evidence

The study’s limitations should be clearly acknowledged. Its retrospective design, single-center setting, modest sample size and selected referral population restrict generalizability. Bone marrow biopsy was performed for clinical reasons in patients with suboptimal response or loss of efficacy, not as systematic surveillance in all treated children. Therefore, the reported prevalence cannot be extrapolated to the broader pediatric ITP population. A further limitation is the absence of systematic baseline bone marrow morphology before TPO-RA initiation. Consequently, pre-existing reticulin deposition or occult marrow abnormalities cannot be excluded retrospectively in every patient, and the association between treatment intensification and MF grade ≥ 2 should not be interpreted as definitive proof of causality. Nevertheless, Ma et al. report that patients underwent comprehensive diagnostic evaluation, including genetic testing when clinically indicated, to exclude congenital thrombocytopenias, secondary ITP and concurrent hematologic malignancy [15]. There is also potential confounding by indication: patients requiring overdose or multiple switches are likely to have more refractory disease, and refractoriness itself may correlate with unmeasured biological factors. The high frequency of avatrombopag use among patients with MF ≥ 2 should likewise be interpreted cautiously, as the authors note that it likely reflects later-line use in refractory patients rather than a direct drug-specific causal effect [15].

## 9. Practical Implications

Despite these limitations, the paper provides a practical message. The danger may not lie in careful, label-concordant use of TPO-RAs, but in therapeutic drift: the gradual normalization of higher-than-approved doses, prolonged treatment escalation and serial switching when response remains inadequate. In pediatric ITP, where treatment goals should include not only platelet count but also long-term marrow safety, this distinction is central.

From a clinical standpoint, the findings support several principles. First, TPO-RAs should be used with clear response targets and predefined stopping or switching rules. Second, dose adjustments within the approved range remain appropriate, but escalation beyond recommended limits should be exceptional, time-limited and explicitly justified. Third, repeated TPO-RA switching in a non-responder should be viewed as a warning sign, not simply as routine sequencing. Fourth, alternative immunomodulatory strategies, including rituximab and, in selected refractory cases, emerging approaches such as anti-CD38 therapy, should be considered before prolonged off-label TPO-RA intensification [17,18,19]. Finally, children exposed to high-intensity TPO-RA strategies may warrant closer hematologic surveillance and a lower threshold for marrow reassessment.

Pending prospective validation, these observations also support a pragmatic surveillance framework for children exposed to long-term or stepwise-intensified TPO-RA treatment. Before starting or escalating TPO-RA therapy, clinicians should re-examine the diagnosis, previous marrow findings if available, family history, platelet size, syndromic features, and potential secondary causes. During treatment, platelet response, bleeding phenotype, full blood count and peripheral smear remain essential, but they should not be considered sufficient to exclude marrow fibrosis in high-risk situations. A repeat marrow evaluation should be considered when off-label dosing above approved pediatric limits is being contemplated or has been maintained, when more than one TPO-RA switch is required because of primary non-response or loss of response, when sustained above-label therapy is prolonged despite inadequate platelet recovery, or when additional warning signs appear, such as new cytopenias, leukoerythroblastosis, splenomegaly, unexplained constitutional symptoms or unexpected loss of response. Such an approach is not intended to mandate routine serial biopsies for all children receiving label-concordant TPO-RA therapy, but to define a lower threshold for marrow reassessment when the treatment pattern itself indicates supraphysiological stimulation.

## 10. Conclusions

The broader lesson is that response-adapted therapy must also be risk-adapted therapy. In pediatric ITP, clinicians often treat not only laboratory thrombocytopenia but also bleeding risk, family anxiety, school participation and the cumulative burden of chronic disease. These realities may understandably push treatment toward escalation. The present study reminds us that the marrow is not a passive target of therapy. It is a dynamic tissue responding to pharmacologic stimulation, and sustained supraphysiological activation may have structural consequences.

Ma and colleagues have therefore provided more than a safety warning. They have proposed a clinically useful framework: distinguish acceptable long-term TPO-RA use in responders from potentially hazardous intensification in non-responders. This distinction should influence future guidelines, registry design and prospective studies. Most importantly, it should influence daily clinical decisions at the bedside.

For the pediatric hematologist, the message is measured but clear: when a child with ITP does not respond to maximum approved TPO-RA dosing, the next step should not automatically be further stimulation. It may be time to change the therapeutic question.

## Figures and Tables

**Table 1:** Approximate annual incidence and prevalence of chronic pediatric ITP in the EU-27 and Italy, using children aged 0–14 years as the denominator.

Area	Children Aged 0–14 Years	New Chronic ITP Cases/Year	Prevalence If Mean Duration = 3 Years	Prevalence If Mean Duration = 5 Years
EU-27	~64.9 million	~325	~970	~1620
Italy	~7.0 million	~35	~105	~175

Note: Estimates are based on a chronic pediatric ITP frequency of 0.5/100,000 children/year. A 10-year mean duration scenario would yield approximately 3240 prevalent children in the EU-27 and 350 in Italy.

## Data Availability

No new datasets were generated or analyzed in this commentary.
